# Geleophysic dysplasia: novel missense variants and insights into ADAMTSL2 intracellular trafficking

**DOI:** 10.1016/j.ymgmr.2019.100504

**Published:** 2019-09-05

**Authors:** Pasquale Piccolo, Valeria Sabatino, Pratibha Mithbaokar, Elena Polishchuck, Simon K. Law, Lorena Magraner-Pardo, Tirso Pons, Roman Polishchuck, Nicola Brunetti-Pierri

**Affiliations:** a Telethon Institute of Genetics and Medicine, Pozzuoli, Italy; b Department of Translational Medicine, Federico II University of Naples, Naples, Italy; c Jules Stein Eye Institute, University of California, Los Angeles, CA, USA; d Spanish National Cancer Research Center (CNIO), Madrid, Spain; e Department of Immunology and Oncology, National Center for Biotechnology, Spanish National Research Council (CNB-CSIC), Madrid, Spain

**Keywords:** Geleophysic dysplasia, ADAMTSL2, Connective tissue disorder, TGF-β signaling

## Abstract

Geleophysic dysplasia (GPHYSD1, MIM231050; GPHYSD2, MIM614185; GPHYSD3, MIM617809) is an autosomal disorder characterized by short-limb dwarfism, brachydactyly, cardiac valvular disease, and laryngotracheal stenosis. Mutations in *ADAMTSL2*, *FBN1*, and *LTBP3* genes are responsible for this condition. We found that three previously described cases of GPHYSD diagnosed clinically were homozygote or compound heterozygotes for five *ADAMTSL2* variants, four of which not being previously reported. By electron microscopy, skin fibroblasts available in one case homozygote for an *ADAMTSL2* variant showed a defective intracellular localization of mutant ADAMTSL2 protein that did not accumulate within lysosome-like intra-cytoplasmic inclusions. Moreover, this mutant ADAMTSL2 protein was less secreted in medium and resulted in increased SMAD2 phosphorylation in transfected HEK293 cells.

## 1 Introduction

Geleophysic dysplasia (GPHYSD1, MIM231050; GPHYSD2, MIM614185; GPHYSD3, MIM617809) presents with short stature, small hands and feet, cardiac valvular disease, hepatomegaly, joint contractures, and thickened skin [1,2]. GPHYSD is caused by bi-allelic mutations in a disintegrin and metalloproteinase with thrombospondin motifs-like 2 (*ADAMTSL2*) [3], heterozygous mutations in fibrillin-1 (*FBN1*) [4] or in latent transforming growth factor β (TGF-β)-binding protein-3 (*LTBP3*) [5]. Mutations in *ADAMTSL2* and *FBN1* affect Transforming Growth Factor-β (TGF-β) signaling [3,4] whereas no evidence of altered TGF-β signaling was detected in serum of patients carrying *LTBP3* variants [5]. TGF-β is sequestered in an inactive, latent form by the extracellular matrix (ECM) through interactions with ECM proteins. Among these, ordered polymers of fibrillin play a major role, as they bind latent TGF-β and maintain TFG-β biologically inactive. ADAMTSL2 belongs to a subgroup of ADAMTS-like proteins sharing homology only with non-enzymatic domains of ADAMTS metalloproteinase [6]. ADAMTSL2 consists of a 30 amino acid signal peptide, an N-terminal thrombospondin type 1 repeat (TSR1), a cysteine rich-module, a peculiar spacer domain, an N-glycan-rich module, six tandem TSRs (TSR2-7), and a C-terminal PLAC (protease and lacunin) domain [7]. ADAMTSL2 binds fibrillin [4,8] but its role in microfibril assembly is not completely understood.Recent findings show that ADAMTSL2 specifically regulates in tendons microfibril assembly that is required for tendon growth [9].

To date, 29 *ADAMTSL2* mutations (18 missense, 4 nonsense, 2 deletions and 5 affecting splicing) have been described in 43 families with GPHYSD but a clear genotype/phenotype correlation has not emerged [3,[10], [11], [12], [13], [14], [15], [16], [17]]. In this study, we identified *ADAMTSL2* variants in three previously published cases [[18], [19], [20]] and provide hints into mutated ADAMTSL2 intracellular trafficking.

## 2 Materials and methods

### 2.1 DNA sequencing and computational analyses

Genomic DNA was extracted from peripheral blood using standard procedures. PCR primers for *ADAMTSL2* amplification were previously described [3]. Amplicons purification and sequencing were performed by PRIMM (Milan, Italy). NM_001145320.1 and NP_001138792.1 were used as *ADAMTSL2* reference sequences for variant reporting.

Three distinct approaches were used to investigate the functional and three-dimensional (3D) structural effect of the missense variants: (1) measurement of the evolutionary conservation in the genome of vertebrate and mammalian species (i.e., phyloP, phastCons, GERP++), (2) prediction of the impact of amino acid substitutions in protein function (i.e., SIFT, Polyphen2, LRT, MutationAssessor, MutationTaster, fathmm-MKL, PROVEAN, MetaSVM, MetaLR, REVEL), and (3) prediction of protein 3D destabilization (i.e., PoPMuSiCv3.1, CUPSAT, I-Mutant v3.0, MAESTRO, INPS-3D). A consensus interpretation was established according to the number of tools predicting a damaging effect versus a benign or tolerated one. The consensus was given if at least 70% (functional impact: 8 of 11; 3D stability: 4 of 5) of predictors agreed in the variant classification. Otherwise, the results were considered as inconclusive. Moreover, we included functional annotations (e.g., ligand binding sites, catalytic residues, post-translational modifications of proteins, residues in protein–protein interaction interfaces) retrieved by the Structure-PPi system [21]. Structure-PPi also considers residues in physical proximity (at a 5Å distance) to amino acid changes found in human diseases. The co-localizing and co-clustering of somatic mutations and germline variants onto protein 3D-structure have been applied to link rare variants with functional consequence [22].

### 2.2 Cell studies

Fibroblast culture was established from skin biopsy. Primary culture fibroblasts were cultured in Dulbecco's Modified Eagle Medium (DMEM) supplemented with 10% Fetal Bovine Serum (FBS). They were then fixed with 4% paraformaldehyde in phosphate buffered saline (PBS) pH 7.4 and covered with 50 mM NH_4_Cl/PBS to reduce quenching. Cells were permeabilized with 0.1% saponin, incubated for 1 h with blocking solution [0.5% bovine serum albumin (BSA), 50 mM NH_4_Cl, 0.1% saponin in PBS pH 7.4]. The primary antibodies used were: anti-ADAMTSL2 (#GTX102069, GeneTex, Irvine, CA) and anti-TGN46 (#AHP500, AbD SeroTec, Oxford, UK). The secondary antibodies were: AlexaFluor-488 anti-rabbit (#A-21206) for ADAMTSL2 and AlexaFluor-594 anti-sheep (#A-11016) for TGN46, all purchased from Life Technologies (Carlsbad, CA, USA). Nuclei were counterstained with diamidino-2-phenylindole (DAPI) (#D-21490, Life Technologies, Carlsbad, CA, USA). Confocal images were obtained using LSM 710 confocal laser scanning microscope and ZEN 2008 software (Carl Zeiss, Oberkochen, Germany). Each experiment was performed in duplicate.

A pCMV-SPORT6 plasmid bearing human *ADAMTSL2* cDNA was purchased from Thermo Fisher Scientific (Waltham, MA, USA). Mutations were introduced using QuickChange XL site-directed mutagenesis kit (Agilent Technologies, Wilmington, DE, USA) according to the manufacturer's instructions and confirmed by direct DNA sequencing. HEK293 cells were transfected with wild-type and mutated *ADAMTSL2* constructs using TransIT-LT1 transfection reagent (Mirus Bio, Madison, WI, USA) according to manufacturer's instructions. 72 h after transfection, media and cells were collected for Western blotting with anti-ADAMTSL2 (#GTX-102069, GeneTex, Irvine, CA, USA) and anti-pSMAD2 (#3108, Cell Signaling Technology, Danvers, MA, USA) antibodies. Anti-Calnexin (#SPA860, Assays Design, Ann Arbor, MI, USA) was used for normalization. Analysis of protein band intensities were performed by Quantity One basic software (Bio-Rad laboratories, Hercules, CA, USA).

### 2.3 Electron microscopy

Fibroblasts were tripsinized and resuspended in glutaraldehyde for electron microscopy (EM). For immuno-EM analysis of ADAMTSL2 distribution, fibroblasts were fixed with a mixture of 4% paraformaldehyde (PFA) and 0.05% glutaraldehyde for 10 min at room temperature, then washed once with 4% PFA to remove residual glutaraldehyde and fixed again with 4% PFA for 30 min at room temperature. Cells were then incubated with blocking/permeabilizing mixture (0.5% BSA, 0.1% saponin, 50 mM NH_4_Cl) for 30 min and next with the primary polyclonal antibody against ADAMTSL2 diluted 1:500 in blocking/permeabilizing solution. The following day, cells were washed and incubated with the secondary antibody, the anti-rabbit Fab fragment coupled to 1.4-nm gold particles (diluted 1:50 in blocking/permeabilizing solution) for 2 h at room temperature. Specimens were post-fixed as previously described [23] and after dehydration they were embedded in epoxy resin and polymerized at 60 °C for 72 h. 60 nm-thin sections were cut at the Leica EM UC7 microtome. EM images were acquired from thin sections using a FEI Tecnai-12 electron microscope equipped with a VELETTA CCD digital camera (FEI, Eindhoven, The Netherlands). Quantification of ADAMTSL2-associated gold particles was performed using iTEM software (Olympus SYS, Germany). Total number of gold particles was counted within the cell and gold particles in the Golgi area reported as percent of total.

### 2.4 Statistical analyses

Statistical significance was computed using the Student's two tail test. A *p* value <.05 was considered statistically significant.

## 3 Results

### 3.1 *ADAMTSL2* variants in three GPHYSD individuals and ultrastructure studies

**Fig. 1:**
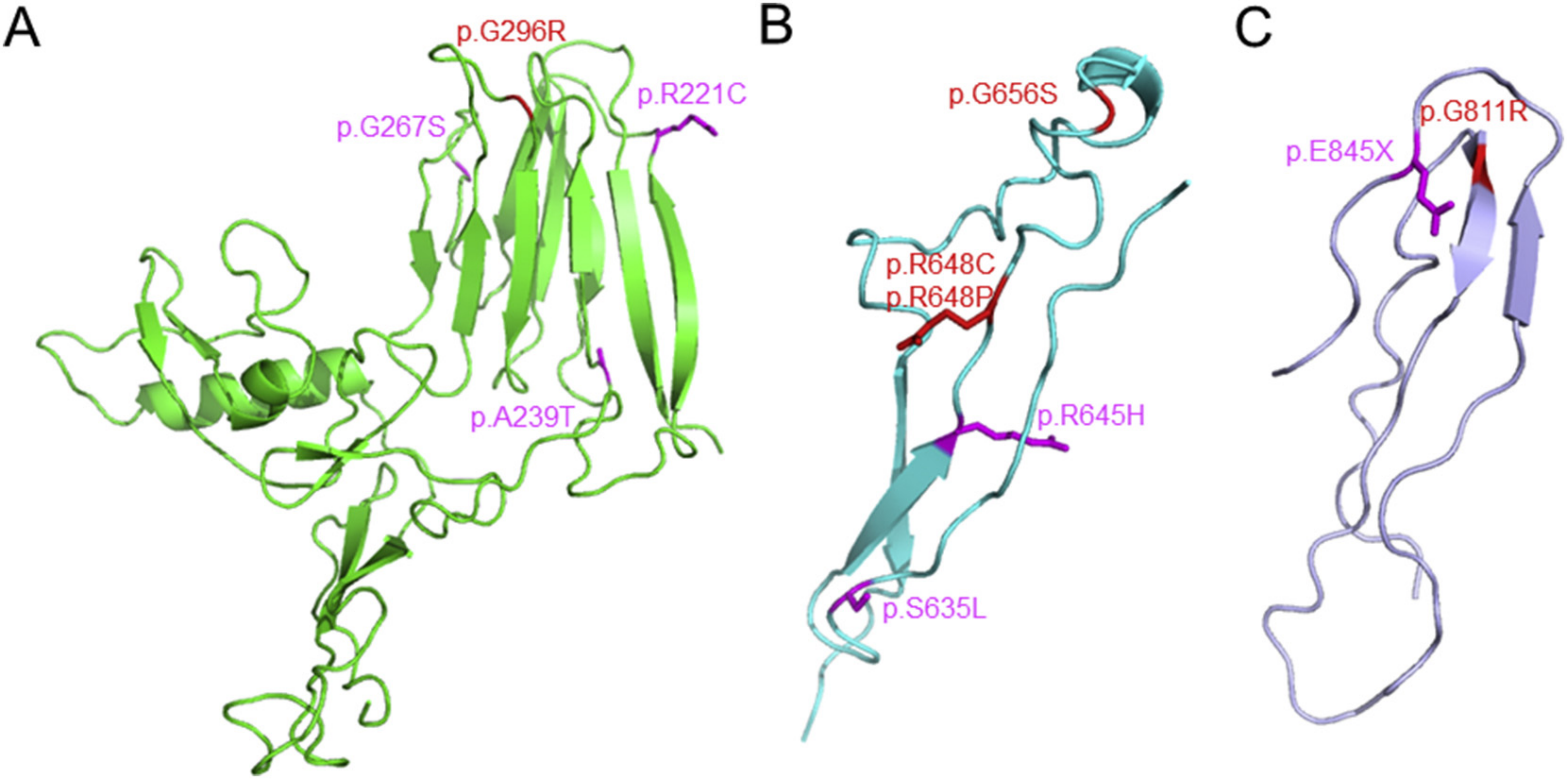
Mapping of *ADAMTSL2* variants is shown in red onto the 3D models of the spacer domain (A) and the TSR3 (B) and TSR6 (C) domains. The positions of previoulsy reported variants in these domains are also shown in magenta. (For interpretation of the references to colour in this figure legend, the reader is referred to the web version of this article.)

Direct *ADAMTSL2* sequencing was performed in three previously published cases. Their clinical features are presented in greater details elsewhere [[18], [19], [20]]. In all three cases, either homozygous or compound heterozygous *ADAMTSL2* variants were identified (Table 1). The variants found in subject 1 c.2431G > A (p.Gly811Arg, rs113994124) localized in the spacer domain and c.1942C > T (p.Arg648Cys, rs1198735320) localized in the TSR3 domain are annotated in dbSNP and 1000Genomes, whereas the remaining variants including c.1943G > C (p.Arg648Pro) and c.1966G > A (p.Gly656Ser) in subject 2 both affecting TSR3, and the homozygous c.886G > A (p.Gly296Arg) variant of subject 3 affecting the spacer domain were not previously reported but they are clustered with two or more other previously reported GPHYSD-related variants on 3D models of ADAMTSL2 (Fig. 1). All five variants are not reported in controls of the Exome Aggregation Consortium (ExAC) and Genome Aggregation Database (gnomAD) databases. However, the region between exons 9 and 18 of *ADAMTSL2* has poor coverage in ExAC and gnomAD databases and better coverage in 1000Genomes. All variants except p.Gly296Arg were predicted to have a damaging effect (impaired functional predictions and destabilization of the 3D structure) (Supplementary Table 1). The variant p.Gly296Arg was classified as inconclusive according to the functional impact predictions and corresponding 3D structural predictions. Nevertheless, all variants affected conserved positions in vertebrate and mammalian species, as shown by phyloP, phastCons, and GERP++.

**Table 1:** Summary of *ADAMTSL2* variants.

Subjects	1^a^	2^b^	3^c^
Gender	M	F	M
*ADAMTSL2* variants allele1/allele2	c.1942C > T(p.Arg648Cys)/ c.2431G > A(p.Gly811Arg)	c.1943G > C(p. Arg648Pro)/ c.1966G > A(p.Gly656Ser)	c.886G > A(p.Gly296Arg)/ c.886G > A(p.Gly296Arg)
EM inclusions	N.D.	N.D.	+

Described in: ^a^Rennie et al. [18]; ^b^Zhang et al. [19]; ^c^Titomanlio et al. [20].

M = male; F = female; N.D. = not determined.

**Fig. 2:**
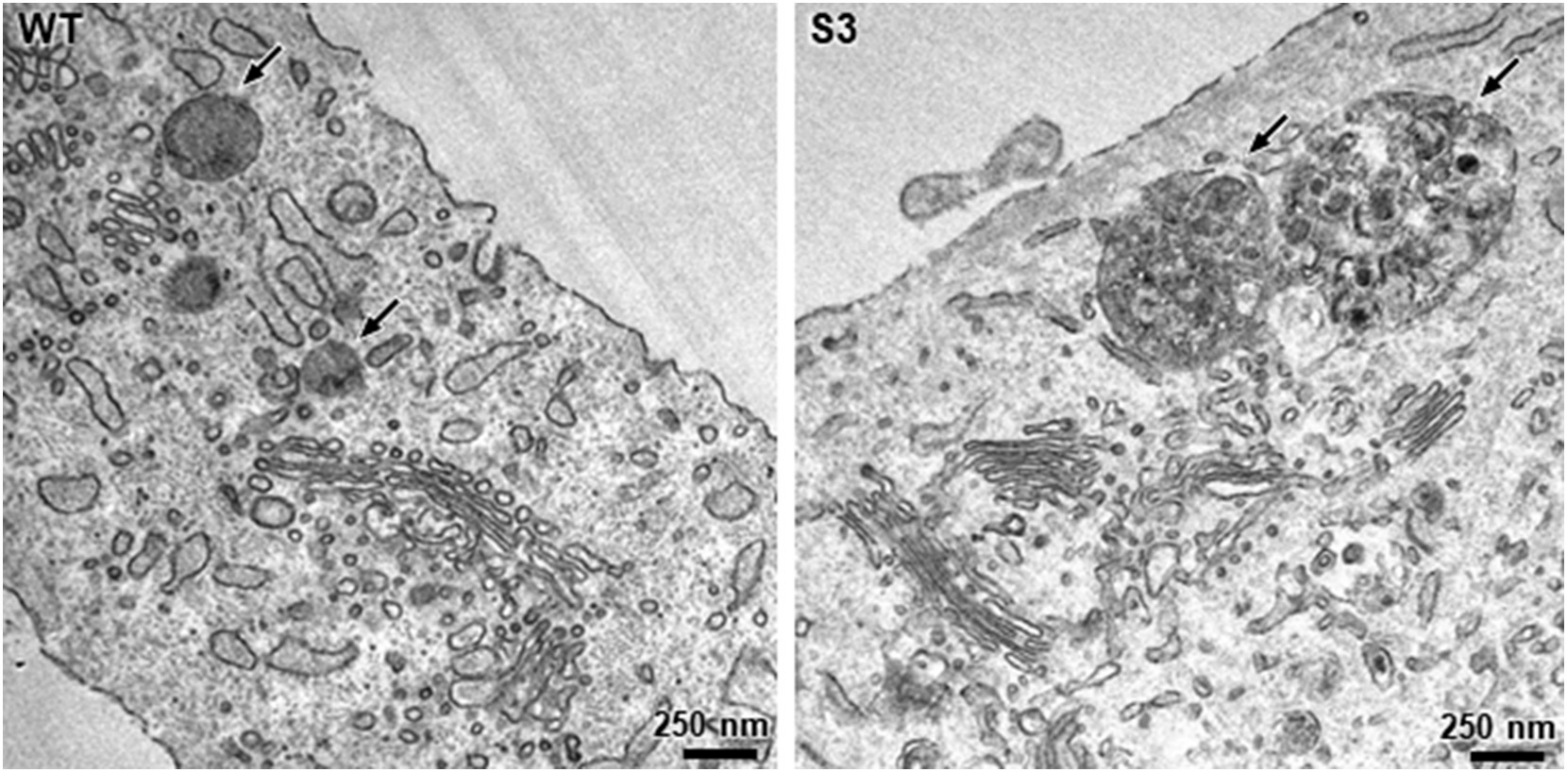
Representative EM images of cultured skin fibroblasts of subject 3 (S3) carrying the p.Gly296Arg variant in *ADAMTSL2* showing intracytoplasmic multilamellar and electron-dense material in lysosomal-like vesicles (arrows). Fibroblast from healthy subject (WT) is shown as control and arrows are here pointing to lysosomes.

Intracellular inclusion bodies were previously reported in GPHYSD cases [2,24,25] and we recently found that storage occurs within lysosomes in GPHYSD cells carrying *FBN1* mutation [26]. Primary fibroblasts from skin biopsy available only for subject 3 were analyzed by EM and showed lysosomal-like vesicles with lamellar structure appearance and electron-dense storage material (Fig. 2) suggesting that inclusions are a feature of GPHYSD cells either carrying *ADAMTSL2* or *FBN1* mutations [26].

### 3.2 ADAMTSL2 carrying the p.Gly296Arg variant fails to traffic through the Golgi complex but does not accumulate in lysosome-like vesicles

**Fig. 3:**
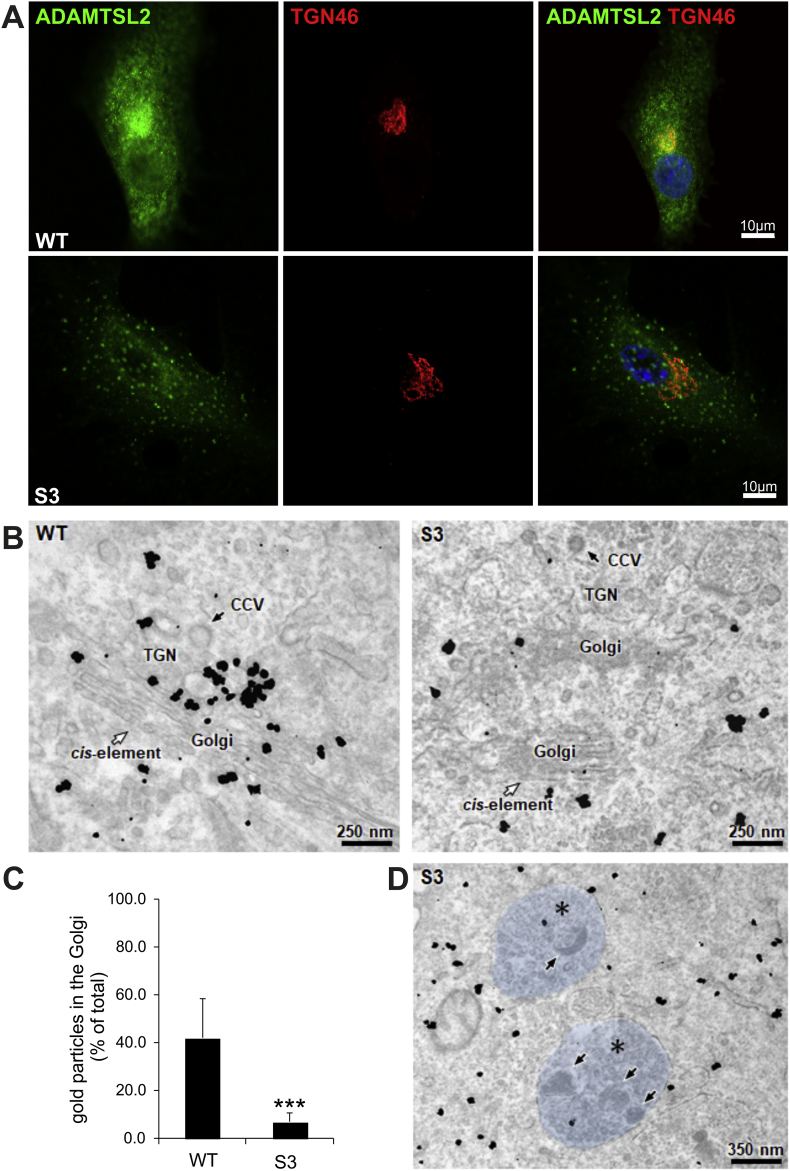
(A) Skin fibroblasts of subject 3 (S3) carrying the homozygous p.Gly296Arg variant show intracellular ADAMTSL2 mislocalization. Representative confocal immunofluorescence images of permeabilized fibroblasts stained for ADAMTSL2 (green) and TGN46 (red), a trans-Golgi cisternae marker. In wild-type (WT) cells, ADAMTSL2 has diffuse cytoplasmatic signal, with a strong positive signal at the Golgi level where co-localization with TGN46 occurs, whereas fibroblasts from S3 display a focal cytoplasmatic distribution of ADAMTSL2 which fails to co-localize with TGN46. Nuclei were counterstained with DAPI (blue). (B) Immunogold staining shows ADAMTSL2 signal in the trans-Golgi network (TGN) in WT fibroblasts but not in fibroblasts from S3. Clathrin-coated vesicles (CCV) are also highlighted. (C) Quantification of anti-ADAMTSL2 gold nanoparticles at the Golgi apparatus in WT and S3 fibroblasts. Total number of gold particles was counted within the cells and gold particles in the Golgi area are reported as percent of total. *t*-test: ***p < .001. (D) Magnified image of anti-ADAMTSL2 immuno-EM in fibroblasts from S3 showing no accumulation of mutant ADAMTSL2 in lysosomal-like vesicles highlighted in light blue and with asterisks. (For interpretation of the references to colour in this figure legend, the reader is referred to the web version of this article.)

ADAMTSL2 is a glycoprotein that traffics through the Golgi complex [7]. To investigate whether mutated ADAMTSL2 is correctly targeted to the Golgi, we performed on GPHYSD fibroblasts from subject 3 and control fibroblasts a confocal immunofluorescence analysis for ADAMTSL2 and TGN46, a trans-Golgi network (TGN) marker [27]. Control fibroblasts showed a punctate cytosolic pattern of ADAMTSL2 signals and a distinctive ADAMTSL2 accumulation around the nuclei, corresponding to TGN46 positive cisternae (Fig. 3A). In contrast, GPHYSD fibroblasts showed reduced ADAMTSL2 staining with distribution in cytosolic clumps lacking TGN46 co-localization (Fig. 3A). To confirm that mutated ADAMTSL2 protein fails to traffic though the Golgi, we performed immuno-EM with anti-ADAMTSL2 antibody in GPHYSD fibroblasts. GPHYSD fibroblasts showed a diffuse cytoplasmic ADAMTSL2 signal in contrast to wild-type fibroblasts exhibiting distribution of ADAMTSL2 in the Golgi apparatus with gold particles concentrated at the TGN area (Fig. 3B–C). Moreover, intracytoplasmic inclusions in GPHYSD fibroblasts did not appear to contain ADAMTSL2-positive signals (Fig. 3D).

### 3.3 The p.Gly296Arg ADAMTSL2 variant impairs protein secretion and results in increased TGF-β signaling

**Fig. 4:**
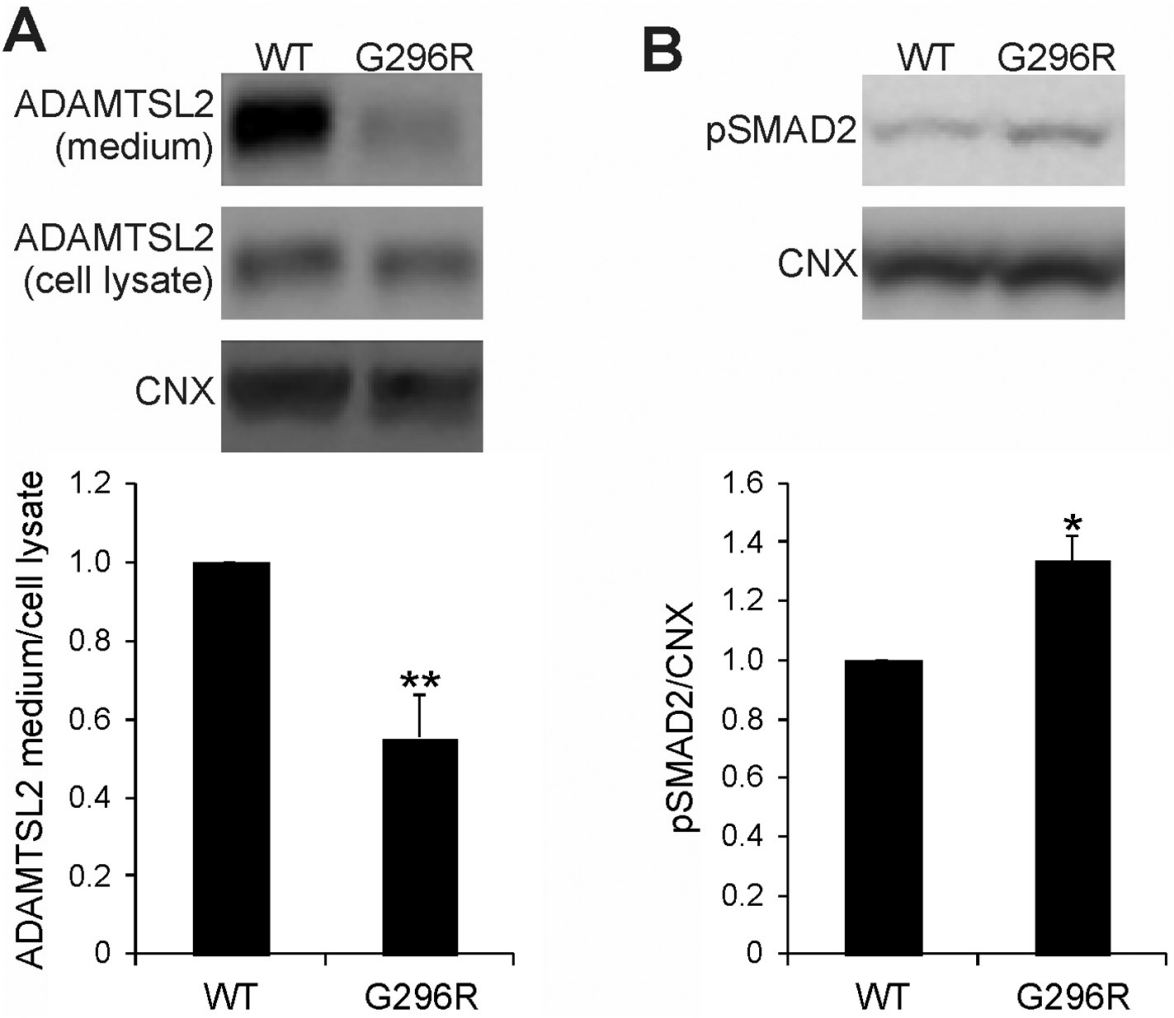
Mutated ADAMTSL2 is poorly secreted and activates TGF-β signaling. (A) Western blotting for ADAMTSL2 on conditioned media and cell lysates of HEK293 cells transfected with wild-type (WT) and mutant ADAMTSL2 constructs. Quantification of ADAMTSL2 in media over cell lysates normalized for calnexin (CNX) is shown (*n* = 6; *t*-test: **p < .01). (B) Western blot of SMAD2 phosphorylation (pSMAD2), a downstream effect of TGF-β pathway activation, in HEK293 cells transfected with mutant or wild-type ADAMTSL2. CNX is shown as loading control (n = 6; *t*-test: *p < .05).

We next investigated the functional consequences of the p.Gly296Arg variant of subject 3 by transient transfection of HEK293 cells with a plasmid bearing the mutated human *ADAMTSL2* cDNA. Conditioned media and cell lysates were analyzed by western blot using an anti-ADAMTSL2 antibody. Consistent with other previously reported mutations [3], reduced levels of ADAMTSL2 protein were detected in conditioned medium of cells transfected with the mutant *ADAMTSL2* construct compared to control cells expressing the wild-type ADAMTSL2 (Fig. 4A). Moreover, lysates of HEK293 cells transfected with mutated *ADAMTSL2* showed increased phosphorylated SMAD2 (pSMAD2) compared to cells transfected with wild-type *ADAMTSL2* (Fig. 4B). Taken together, these findings show that the p.Gly296Arg variant results in poor secretion of ADAMTSL2 and increased TGF-β signaling in transfected HEK293 cells.

## 4 Discussion

In this study, we identified five novel *ADAMTSL2* variants in three published cases of GPHYSD [[18], [19], [20]]. Storage material staining positively for periodic acid-Schiff (PAS) staining in GPHYSD patients, has previously suggested glycoprotein accumulation [2,24]. Inclusions have been detected in multiple tissues including skin, liver, bone cartilage, trachea, and heart, and in primary skin fibroblasts. These inclusions have been described as lysosomal-like vacuoles containing granular material, lamellar structures or electron-dense material [[14], [28], [29], [30]]. Similar inclusions were also found in bronchial epithelial cells of *Adamtsl2*^−^^/^^−^ mouse, thus suggesting glycogen storage [8]. Moreover, we previously found that inclusions are also present in fibroblasts of GPHYSD patients carrying *FBN1* mutations and Myhre syndrome (MIM139210) patients carrying *SMAD4* mutations [26], suggesting that a common pathway is responsible for the formation of such inclusions. Here, we showed that lysosomal inclusions do not appear to contain mutant ADAMTSL2.

We also showed that ADAMTSL2 with the missense variant p.Gly296Arg fails to localize in the Golgi and its secretion is impaired in contrast to wild-type ADAMTSL2 that traffics trough the Golgi complex and is efficiently secreted. Therefore, a combination of ADAMTSL2 secretion and pSMAD2 analyses as performed in this study in cell lines might be useful for validation of the pathogenicity of *ADAMTSL2* variants without the need of obtaining skin fibroblasts. Moreover, these cell assays may be useful to search for drugs increasing ADAMTSL2 secretion which might have therapeutic potential. However, further studies on ADAMTSL2 degradative pathways can define the fate of mutated protein and its consequence on TGF-β signaling.

## URLs

dbSNP: https://www.ncbi.nlm.nih.gov/snp/

ClinVar: https://www.ncbi.nlm.nih.gov/clinvar/

1000Genome Project: http://www.1000genomes.org/

ExAC Browser: http://exac.broadinstitute.org/

gnomAD: http://gnomad.broadinstitute.org/

dbNSFP: https://sites.google.com/site/jpopgen/dbNSFP

Structure-PPi: http://rbbt.bsc.es/Structure

PoPMuSiCv3.1: http://dezyme.com

CUPSAT: http://cupsat.tu-bs.de

I-Mutant v3.0: http://gpcr.biocomp.unibo.it/cgi/predictors/I-Mutant3.0/I-Mutant3.0.cgi

MAESTRO: https://biwww.che.sbg.ac.at/maestro/web/

INPS-3D: http://inpsmd.biocomp.unibo.it/inpsSuite/default/index3D

## Appendix A Supplementary data

Supplementary material Image 1
