# Supplementary material for: Geleophysic dysplasia: novel missense variants and insights into ADAMTSL2 intracellular trafficking
Source: Mol Genet Metab Rep. 2019 Sep 5;21:100504. doi: 10.1016/j.ymgmr.2019.100504 (PMC6732760; doi:10.1016/j.ymgmr.2019.100504)
Supplement: Supplementary file 1 — Supplementary material [file mmc1.docx]

**Online Supplementary Material**

**Title:** Geleophysic dysplasia: novel missense variants and insights into ADAMTSL2 intracellular trafficking.

**Authors:** Pasquale Piccolo^1,2^, Valeria Sabatino^1,^ **^¶^**, Pratibha Mithbaokar^1^, Elena Polishchuck^1^, Simon K. Law^3^, Lorena Magraner-Pardo^4^, Tirso Pons^5^, Roman Polishchuck^1^, and Nicola Brunetti-Pierri^1,2,*^

**Institutions:** ^1^Telethon Institute of Genetics and Medicine, Pozzuoli, Italy; ^2^Department of Translational Medicine, Federico II University of Naples, Naples, Italy; ^3^Jules Stein Eye Institute, University of California, Los Angeles, CA, USA; ^4^Spanish National Cancer Research Center (CNIO), Madrid, Spain; ^5^Department of Immunology and Oncology, National Center for Biotechnology, Spanish National Research Council (CNB-CSIC), Madrid, Spain.

***Corresponding author:** Nicola Brunetti-Pierri, M.D.

Telethon Institute of Genetics and Medicine

Via Campi Flegrei 34

Pozzuoli (NA), 80078 Italy

Phone: +39 081 19230661

Fax: +39 081 5609877

E-mail: [brunetti@tigem.it](mailto:brunetti@tigem.it)

**Supplementary Table 1.** Functional annotation and interpretation of *ADAMTSL2* variants.

| **Genomic location** | **cDNA**  **Protein** | **3D structure** | **dbSNP/ClinVar** | **Evolutionary**  **conservation (a)** | **Protein function prediction (a)** | **3D structural prediction and functional annotations (b)** |
| --- | --- | --- | --- | --- | --- | --- |
| chr9:133547160 | c.886G>A  p.G296R | 3D-model by  ModBase:  a.a. 50-333  Position in the 3D structure: ADAM-TS spacer-1 domain | dbSNP: n.a.  ClinVar: n.a. | phyloP (vertebrate): 9.847  phyloP (mammalian): 1.176  phastCons (vertebrate): 1.000  phastCons (mammalian): 0.659  GERP++: 5.16 | SIFT: 0.001 (D)  Polyphen (HDIV): 1.0 (D)  Polyphen (HVAR): 0.997 (D)  LRT_score: 0.002157 (N)  Mutation Taster: 1 (D)  MutationAssessor: 1.73 (N)  fathmm-MKL: 0.987 (D)  PROVEAN: -4.07 (D)  MetaSVM: -0.729 (N)  MetaLR: 0.272 (N)  REVEL: 0.5 (D) | PoPMuSiCv3.1: -0.13 (S)  CUPSAT: -0.97 (D)  I-Mutant v3.0: -1.0 (D)  MAESTRO: -0.210 (S)  INPS-3D: 0.078 (S)  Structure-PPi: neighbor positions in 3D mutated in Kidney (R247T), Colorectal (G296R) and Gastric (E294D) carcinomas |
| **Interpretation** |  | | **Inconclusive** | **Conserved position** | **Inconclusive (7/11)** | **Inconclusive (2/5)** |
| chr9:133568340 | c.1942C>T  p.R648C | 3D model by  I-TASSER:  a.a. 622-686  Position in the 3D structure: TSP type-1 repeat, domain 3 | dbSNP: rs1198735320  ClinVar: n.a. | phyloP (vertebrate): 7.454  phyloP (mammalian): 1.03  phastCons (vertebrate): 1.000  phastCons (mammalian): 0.978  GERP++: 3.25 | SIFT: 0.005 (D)  Polyphen (HDIV): 1.0 (D)  Polyphen (HVAR): 0.992 (D)  LRT_score: 0.000025 (U)  Mutation Taster: 1.0 (D)  MutationAssessor: 3.3 (D)  fathmm-MKL: 0.97079 (D)  PROVEAN: -5.52 (D)  MetaSVM: 0.0061 (D)  MetaLR: 0.4252 (N)  REVEL: 0.44 (N) | PoPMuSiCv3.1: -0.02 (S)  CUPSAT: -0.6 (D)  I-Mutant v3.0: -0.56 (D)  MAESTRO: 0.094 (D)  INPS-3D: -0.226 (D)  Structure-PPi: neighbor positions in 3D mutated in Liver (C649Y) and Thyroid (W650C) carcinomas |
| **Interpretation** |  | | **Inconclusive** | **Conserved position** | **Impaired (8/11)** | **Destabilize (4/5)** |
| chr9:133568341 | c.1943G>C  p.R648P | 3D model by  I-TASSER:  a.a. 622-686  Position in the 3D structure: TSP type-1 repeat, domain 3 | dbSNP: n.a.  ClinVar: n.a. | phyloP (vertebrate): 9.450  phyloP (mammalian): 1.18  phastCons (vertebrate): 1.000  phastCons (mammalian): 0.993  GERP++: 3.25 | SIFT: 0.021 (D)  Polyphen (HDIV): 0.605 (D)  Polyphen (HVAR): 0.597 (D)  LRT_score: 0.000025 (U)  Mutation Taster: 1.0 (D)  MutationAssessor: 3.3 (D)  fathmm-MKL: 0.986 (D)  PROVEAN: -5.03 (D)  MetaSVM: -0.401 (N)  MetaLR: 0.305 (N)  REVEL: 0.5 (D) | PoPMuSiCv3.1: 1.14 (D)  CUPSAT: -1.68 (D)  I-Mutant v3.0: -0.35 (D)  MAESTRO: 0.693 (D)  INPS-3D: -1.216 (D)  Structure-PPi: neighbor positions in 3D mutated in Liver (C649Y) and Thyroid (W650C) carcinomas |
| **Interpretation** |  | | **Inconclusive** | **Conserved position** | **Impaired (8/11)** | **Destabilize (5/5)** |
| chr9:133568364 | c.1966G>A  p.G656S | 3D model by  I-TASSER:  a.a. 622-686  Position in the 3D structure: TSP type-1 repeat, domain 3 | dbSNP: n.a.  ClinVar: n.a. | phyloP (vertebrate): 9.450  phyloP (mammalian): 1.176  phastCons (vertebrate): 1.000  phastCons (mammalian): 0.999  GERP++: 3.25 | SIFT: 0.01 (D)  Polyphen (HDIV): 0.999 (D)  Polyphen (HVAR): 0.993 (D)  LRT_score: 0.0 (U)  Mutation Taster: 1.0 (D)  MutationAssessor: 2.31 (D)  fathmm-MKL: 0.983 (D)  PROVEAN: -3.71 (D)  MetaSVM: -0.22 (N)  MetaLR: 0.435 (N)  REVEL: 0.5 (D) | PoPMuSiCv3.1: 0.38 (D)  CUPSAT: 0.8 (S)  I-Mutant v3.0: -1.59 (D)  MAESTRO: 0.536 (D)  INPS-3D: -0.828 (D)  Structure-PPi: Liver (C649Y) and Thyroid (W650C) carcinomas |
| **Interpretation** |  | | **Inconclusive** | **Conserved position** | **Impaired (8/11)** | **Destabilize (4/5)** |
| chr9:133570346 | c.2431G>A  p.G811R | 3D model by  I-TASSER:  a.a. 797-851  Position in the 3D structure: TSP type-1 repeat, domain 6 | dbSNP:  rs113994124  ClinVar: Pathogenic | phyloP (vertebrate): 9.533  phyloP (mammalian): 1.176  phastCons (vertebrate): 1.000  phastCons (mammalian): 0.1  GERP++: 3.8 | SIFT: 0.0 (D)  Polyphen (HDIV): 0.987 (D)  Polyphen (HVAR): 0.847 (D)  LRT_score: 0.0 (U)  Mutation Taster: 1.0 (D)  MutationAssessor: 3.925 (D)  fathmm-MKL: 0.985 (D)  PROVEAN: -6.33 (D)  MetaSVM: 0.673 (D)  MetaLR: 0.638 (D)  REVEL: 0.8 (D) | PoPMuSiCv3.1: 1.63 (D)  CUPSAT: -1.27 (D)  I-Mutant v3.0: -0.49 (D)  MAESTRO: 1.319 (D)  INPS-3D: -0.387 (D)  Structure-PPi: neighbor positions in 3D mutated in Thyroid (E836K) and Liver (E845K) carcinomas |
| **Interpretation** |  | | **Pathogenic** | **Conserved position** | **Impaired (10/11)** | **Destabilize (5/5)** |

(a) The scores were extracted from dbNSFP v4.0. The larger the score, the more conserved the site. Scores range from: phyloP100way_vertebrate [phylogenetic p-values; -20.0 to 10.003]; phyloP20way_mammalian [phylogenetic p-values; -13.282 to 1.199]; phastCons100way_vertebrate and phastCons20way_mammalian [score based on the multiple alignments; 0 to 1]; GERP++ [RS score; -12.3 to 6.17]. Deleterious functional impact (D), Neutral (N), and Unknown (U); (b) Values are in kcal/mol, Destabilizing (D) and Stabilizing (S). ADAMTSL2: UniProt accession number Q86TH1 and Ensembl Protein IDs: ENSP00000346478, ENSP00000376780. n.a.: not annotated variant.
